# Investigating the use and awareness of artificial sweeteners among diabetic patients in Bangladesh

**DOI:** 10.1371/journal.pone.0295272

**Published:** 2023-12-13

**Authors:** Aparna Shil, Jufen Zhang, Havovi Chichger

**Affiliations:** 1 Department of Botany, Jahangirnagar University, Dhaka, Bangladesh; 2 Clinical Trial Unit, School of Medicine, Anglia Ruskin University, Cambridge, United Kingdom; 3 Biomedical Research Group, School of Life Science, Anglia Ruskin University, Cambridge, United Kingdom; Zagazig University Faculty of Human Medicine, EGYPT

## Abstract

**Background:**

As with many countries around the world, the incidence of diabetes in Bangladesh is increasing significantly. Whilst there is controversy in the field regarding the health impact of artificial sweeteners in Western communities, the link between sweetener consumption and awareness in Bangladesh has not been established.

**Methods:**

In the present study, 260 diabetic patients completed a questionnaire survey to investigate the use and awareness of sweeteners and how this links to demographics and potential co-morbidities.

**Results:**

Findings show that daily artificial sweetener consumption is significantly associated with hypertension but not other co-morbidities such as kidney disease or obesity. We further demonstrate that there is limited checking of artificial sweeteners in food or drink products by participants. the rurality of diabetic participants was found to significantly correlates with lower awareness of any health impact of artificial sweeteners.

**Conclusions:**

The findings from this study demonstrate that there is a need to increase the awareness of artificial sweetener use in diabetic patients in Bangladesh. Combined with a more robust understanding of the health impact of artificial sweeteners, these findings suggest that there is potential to improve outcomes for diabetic patients by improving this awareness.

## Introduction

Diabetes is responsible for 6.7 million deaths worldwide and is estimated to be the 7^th^ leading reason for global death by 2030 [1]. In 2021 global prevalence of diabetes in adults was 537 million, which is estimated to increase to 10.5% by 2045 [1]. In Southeast Asia, diabetes is observed in a younger population than in Western countries however the link between dietary habits, lifestyle and diabetes is well-established around the world [2]. Among the Southeast Asian countries, Bangladesh had a prevalence of diabetes of 14.2% in 2021 with an estimation of increase to 15.3% by 2045, making it one of the top ten countries with numbers of people living with diabetes [3, 4]. Worryingly, this trend is increasing in Bangladesh with the number of pre-diabetics and diabetics increasing 2.5-fold compared to 10 years ago [5, 6]. Furthermore, this increasing population is associated with significant health concerns and co-morbidities. Vascular complications are the leading reason for diabetes-related morbidity and mortality in patients with macrovascular complications, including cardiovascular, peripheral vascular disease, stroke, and microvascular complications such as diabetic kidney disease, retinopathy and neuropathy, the primary vascular concerns [7]. In addition, increasing evidence suggests that diabetes has a causative link with other diseases such as cancers, infections, dementia, and liver diseases [8–10]. Several studies also show that diabetes exacerbates major infectious diseases, such as tuberculosis and malaria, which are predominant in Southeast Asia [11, 12]. Whilst Western countries have significantly improved the treatment options and diabetic monitoring for patients in the past decade, Bangladesh does not have the same level of support and management for patients and the financial and social burden of diabetes has a dramatic impact on the quality of life of patients [2]. There is therefore a significant need to understand the diabetic population in Bangladesh to better assess treatment and management of the condition in these patients.

Since diabetes is closely related with risk factors, such as dietary habits and a sedentary lifestyle, public awareness can effectively prevent the increasing prevalence of this metabolic disease and its associated effects. Investigation of the awareness of diet and lifestyle in diabetic patients has previously focused largely on knowledge, attitude and practice [13]. Traditional diabetes management depends on a patient’s self-care in daily life, that is, changing their diet to reduce consumption of refined sugars [2]. It is now well-understood that other aspects of the diet can be modified to improve outcomes for diabetes patients, including reduced consumption of high-fat foods and increased consumption of dietary fibre. This is due, in large part, to their impact on the composition and function of the gut microbiota [14–17]. We and others have shown the artificial sweetener consumption is strongly linked to disruption of the gut microbiota leading to health conditions related to pre-diabetes [18–20]. This has led to re-consideration of the health concerns linked to the consumption of artificial sweeteners, which would previously have been considered a good sugar-free option for diabetics. There is, however, still controversy in the field regarding the benefits versus negative effects of sweeteners on public health. Whilst the Western population are becoming aware of the need to investigate the effects of artificial sweeteners in the diet on health, with investigator groups such as the SWEET EU Consortium forming [21], many areas of the world are not yet considering their effect on public health. Those studies which do consider public health show conflicting results with some findings indicating that high artificial sweetener consumption is associated with increased all-cause mortality, coronary artery disease and cancer risk, whilst others show no significant link between sweetener consumption and poor health outcomes [22–26]. With the number of diabetics significantly increasing in Bangladesh, in the present study we sought to investigate how this population use artificial sweeteners, and their awareness of these additives, following diagnosis.

## Methods

### Study participants and sampling

The total number of participants studied was 260. Participants had to be over 18 years of age, diagnosed with diabetes and living in Bangladesh. The total number of participants exceeded the required sample size (167) which was based on the Jisc Sample Size calculator with prevalence of diabetes in adults in Bangladesh, 5% precision error and 5% type 1 (α) error [1].

Between July 2021 and February 2022, participants were invited to take part in the study, run on the Jisc tool, through social media (Twitter, Facebook), online adverts, emails, and word of mouth. Voluntary response sampling was used where diabetic patients who saw or heard about the study could directly access the Jisc tool weblink and start the survey themselves. Diabetes patients were not recruited by through attendance at hospitals or clinics and no set sector of the population, other than adults with diabetes living in Bangladesh, were targeted. Participants provided their informed consent after reading the information sheet which was provided in both Bengali and English language.

### Type of study and tools of data collection

A cross-sectional study was used to sample patients with diabetes for study. A structured online questionnaire was adapted from existing literature [27, 28] to examine diabetes demographics, history, and complications as well as habits including dietary (S1 Table). All questions were reviewed and agreed upon by two of the authors (HC and AS) and were pre-tested on 10 people with diabetes, not included in the study, to ensure test-retest showed excellent reproducibility. Internal consistency, measured using Cronbach’s alpha, for different questions was 0.91–0.98 and thus rated as excellent. Before completing the survey, individuals were asked three screener questions to confirm if they were currently aged ≥18 years old, lived in Bangladesh, and had been diagnosed with diabetes. Prospective participants were only able to complete the survey by answering ‘Yes’ to all the screener questions. Where participants were not able to complete the survey themselves (e.g. not able to use a computer), responses were collected via face-to-face conversation and telephone interview with a member of the research team (Dr Shil). Where needed, local language (Bengali) was used in the interview or family members who were able to use a computer completed the survey on behalf of the participant. In both cases, informed consent was given and reported prior to the start of the survey. Participant information was input into the Jisc tool anonymously and, as such, authors do not have access to identifying characteristics. Participants were advised that their responses were input anonymously.

### Ethics

Written informed consent was obtained from participants. Participants were treated in accordance with applicable ethical guidelines that followed the tenets of the Helsinki Declaration. Information that could identify individual participant during or after data collection was not recorded. The study protocol was approved by Anglia Ruskin University’s School of Life Science Research Ethics Panel [ref. no.: BS SREP21-31] and the Biosafety, Biosecurity, and Ethical Committee, Faculty of Biological Sciences at Jahangirnagar University [ref. no.: BBEC, JU/M 2021/7(1)]. There were no deviations from the study protocol after approval was obtained Additional information regarding the ethical, cultural, and scientific considerations specific to inclusivity in global research is included in the Supporting Information (S1 Checklist).

### Statistical analysis

Data was collated on Excel and statistical analysis performed using GraphPad (ver 7.0) for Fisher’s exact test. Logistic regression models were used to evaluate associations and odds ratio (OR) with 95% confidence intervals used to show the results.

## Results

### Participant demographics

The survey was distributed among adults in Bangladesh with diagnosed diabetes and 82.5% of the participants responded online. The demographics were collected (Table 1) and the study population consisted of a total of 260 diabetic patients with 150 males (57.69%) and 110 females (42.31%). Patients from a range of age groups responded to the survey with the majority within the 51–70 years range (54.62%) and 31–50 years range (22.46%). There was a relatively even distribution between responders from village or rural (35.77%), town or suburb (31.92%) and city or urban (32.31%) location. Most participants were diagnosed with type 2 diabetes (85%) with only 8.08% diagnosed with type 1 and 1.54% with gestational diabetes (Table 1). The majority of patients had been diagnosed with diabetes for at least a year (95.38%) with nearly a third diagnosed more than 10 years ago (31.54%). Overall participants reported a fair (57.31%) or good (33.46%) knowledge of diabetes.

**Table 1:** Description of patient demographics. All participants completed questions so % was calculated based on 260 cohort size.

Category	Demographic	No. of participants	%
**Gender**	Male	150	57.69
	Female	110	42.31
**Diabetes condition**	Type 1 diabetes	21	8.08
	Type 2 diabetes	221	85
	Gestational diabetes	4	1.54
	Type of diabetes not known	14	5.38
**Age**	18–30	9	3.46
	31–50	87	22.46
	51–70	142	54.62
	71–90	22	8.46
**Location**	Village/rural	93	35.77
	Town/Suburbs	83	31.92
	City/urban	84	32.31
**Time since diabetes diagnosis**	Less than 1 year ago	12	4.62
	1–3 years ago	56	21.54
	4–10 years ago	110	42.31
	More than 10 years ago	82	31.54
**Knowledge of diabetes**	Good	87	33.46
	Fair	149	57.31
	Poor	24	9.23

### Participant health

Participants were asked to report other health conditions, alongside diabetes, which they suffered from. Most patients noted 3 or less co-morbidities (95.77%) with an even distribution between male and female participants (Table 2A). The most common co-morbidities observed among the participants were hypertension (36.39%), kidney disease (13.01%) and eye disease or condition (11.08%). Interestingly, there were gender differences observed for obesity and kidney disease with a significant increase in female participants reporting obesity and kidney disease compared to male participants (Table 2B). There was also a a significant association observed between the number of co-morbidities and age of participants (z = 2.78, p = 0.005) but not between the number of co-morbidities and gender (z = -0.85, p = 0.395).

**Table 2:** Patient health history. **a)** Number of co-morbidities, calculated from numbers selected by participants in the survey, based on gender; **b)** type of co-morbidity, as reported by participants, based on gender.

**a)**									
**# of co-morbidities**	**All participants**			**Male participants**			**Female participants**		
	**Number**	**%**		**Number**		**%**	**Number**		**%**
0	54	20.77		35		23.33	19		17.27
1	100	38.46		62		41.33	38		34.55
2	72	27.69		37		24.67	35		31.82
3	23	8.85		13		8.67	10		9.09
4	7	2.69		1		0.67	6		5.45
5	2	0.77		1		0.67	1		0.91
6	2	0.77		1		0.67	1		0.91
**b)**									
**Co-morbidity**		**All participants**		**Male participants**			**Female participants**		**Fisher’s exact test** (male vs female)
		**Number**	**%**	**Number**	**%**		**Number**	**%**	
Hypertension		151	36.39	86	38.57		65	34.21	0.778
Obesity		39	9.40	13	5.83		27	14.21	4E-04*
Kidney disease		54	13.01	22	9.87		32	16.84	0.004*
Eye disease/condition		46	11.08	27	12.11		19	10.00	0.88
Gastrointestinal problems		28	6.75	18	8.07		10	5.26	0.457
Foot or leg problems		14	3.37	8	3.59		6	3.16	0.966
Sexual problems		7	1.69	7	3.14		1	0.53	0.084
Dental problems		9	2.17	3	1.35		6	3.16	0.133

* denotes a significant difference between gender (p<0.05).

### Use and awareness of sweeteners by participants

We next sought to understand the consumption of sugars and sweeteners by participants (Table 3A) and their awareness of the health impacts of sweeteners (Table 3B). Extrapolations were made from the questions ‘since being diagnosed with diabetes, how often do you eat sugary foods or artificial sweeteners?’ to provide a change in diet since diagnosis metric in Table 3A. Where a change in consumption was noted, it was designated as an increase i.e. from never consuming sugary foods to sometimes consuming artificial sweeteners, or decrease i.e. from often consuming sugary foods to rarely consuming artificial sweeteners. With regards to frequency of consumption since diagnosis of diabetes, most participants reported consuming sugars 1–2 times per month or less with only 9.62% consuming sugars often (1–2 times per week) and 5% consuming sugars on a daily basis. In contrast, sweetener consumption is more decisively split with nearly half the participants (48.85%) reporting daily consumption of sweeteners, 15% reporting often consuming sweeteners (1–2 times per week) and 22.69% of participants stating they never consume sweeteners. These changes in habits can also be seen with a difference in dietary preference from pre-diagnosis to post-diagnosis with 63.38% of participants shifting from often sugar to often sweetener consumption. With regards to how sweeteners are consumed, there is a spread across different types with the majority of participants (46.63%) noting Zero-Cal as the sweetener which they most commonly consumed. There was an even distribution of sweetener consumption in tea and coffee and half the participants reported drinking sugar-free soft drinks (Table 3A).

**Table 3:** Use and awareness of sweeteners and sugar in the diet. **a)** the frequency of consumption, type of sweeteners and mode of consumption in the diet, as reported by participants, pre- and post-diagnosis of diabetes; **b)** awareness of sweeteners with regards to foods containing sweeteners and their impact on health as reported by participants.

**a)**			
**Category**	**Response**	**No. of participants**	**%**
Frequency of sugary food consumption	Daily (about 1–2 times per day)	13	5
	Often (about 1–2 times per week)	25	9.62
	Sometimes (about 1–2 times per month)	85	32.69
	Rarely (about 1–2 times per year)	77	29.62
	Never (I don’t eat any)	60	23.08
Frequency of artificial sweetener consumption	Daily (about 1–2 times per day)	127	48.85
	Often (about 1–2 times per week)	39	15
	Sometimes (about 1–2 times per month)	22	8.46
	Rarely (about 1–2 times per year)	13	5
	Never (I don’t eat any)	59	22.69
Change in diet, since diagnosis, from sugar to sweetener consumption	Increased sweetener consumption compared to sugar (e.g. rarely sugar to often sweetener)	170	68.38
	Decreased sweetener consumption compared to sugar (e.g. sometimes sugar to never sweetener)	55	21.15
	No change (e.g. rarely sugar to rarely sweetener)	35	13.46
Artificial sweetener types consumed	Sweet n Low	34	9.55
	Splenda No Calorie sweetener	4	1.12
	Canderel Low Calorie sweetener	54	15.17
	Equal sweetener	24	6.74
	Zerocal	166	46.63
	Sugar free Gold	3	0.84
	Other	8	2.25
Use of sweetener in tea/coffee	Yes	97	37.31
	No	114	43.85
	Not applicable (don’t drink tea/coffee)	49	18.85
Type of soft drink consumed	With sugar	79	30.38
	With low sugar	49	18.85
	With no sugar	132	50.77
**b)**			
**Category**	**Response**	**No. of participants**	**%**
Awareness of food containing artificial sweeteners	Yes	119	45.77
	No	141	54.23
Check label for presence of artificial sweeteners	Yes	57	21.92
	No	203	78.08
Awareness of health issues linked to artificial sweeteners	Yes	54	20.77
	No	206	79.23

The self-reported awareness of sweeteners in participants showed an even split between those who are and are not aware of whether sweeteners are present in different foods however the majority (78.08%) noted that they did not specifically check food packaging for sweetener content. Similarly, most of the participants (79.23%) were not aware of any health issues linked to artificial sweeteners (Table 3B).

### Link between sweetener use and awareness with health and demographics

The participant health and demographic data was analysed to assess the association between sweetener use and awareness. Participants who consume sweeteners on a daily basis demonstrate a higher risk of hypertension compared to those who never consume sweeteners (Table 4). Interestingly, there was a close association, though not significant, between participants who often or daily consumed sweeteners and gastrointestinal problems. There was no link between sweetener consumption and other co-morbidities. Interestingly, there was also no link between the use or awareness of sweeteners and time since diagnosis, suggesting that those with diabetes for a longer period did not have a change in behaviour towards or understanding of sweeteners (data not shown).

**Table 4:** Association of sweetener consumption with different types of comorbidity. univariate binary logistic regression models to show the association between the frequency of sweetener consumption and reported co-morbidities.

Co-morbidity	Odds ratio (95% confidence intervals)				
	Never*	Rarely	Sometimes	Often	Daily
Hypertension	1	2.67(0.74–9.63) P = 0.13	1.71(0.63–4.62) P = 0.29	1.38(0.61–3.11) 0.43	2.09(1.11–3.91) 0.021*
Obesity	1	0.53(0.06–4.67) P = 0.57	1.87(0.54–6.51) P = 0.32	0.34(0.07–1.72) P = 0.19	1.48(0.62–3.53) P = 0.37
Kidney disease	1	0.36(0.04–3.10) P = 0.36	0.44(0.09–2.15) P = 0.31	1.31(0.48–3.53) P = 0.59	1.41(0.65–3.04) P = 0.38
Eye disease/condition	1	0.62(0.07–5.52) P = 0.67	1.65(0.43–6.31) P = 0.46	1.63(0.52–5.06) P = 0.40	2.01(0.82–4.91) P = 0.13
Gastrointestinal problems	1	0.36(0.04–3.10) P = 0.35	0.44(0.09–2.14) P = 0.31	0.23(0.05–1.13) P = 0.07	0.45(0.19–1.10) P = 0.08
Foot or leg problems	1	3.39(0.51–22.74) P = 0.21	0.89(0.09–9.03) P = 0.92	1.01(0.16–6.33) P = 0.99	0.92(0.22–3.84) P = 0.91
Sexual problems	1	-	-	1.01(0.16–6.33) P = 0.99	0.45(0.09–2.31) P = 0.34
Dental problems	1	-	-	1.56(0.30–8.13) P = 0.60	0.45(0.09–2.31) P = 0.34
Other	1	-	0.82(0.09–6.98) P = 0.85	-	-

*denotes a significant association <0.05. “-“: No events in the categorical level or omitted due to collinearity.

**Fig 1:**
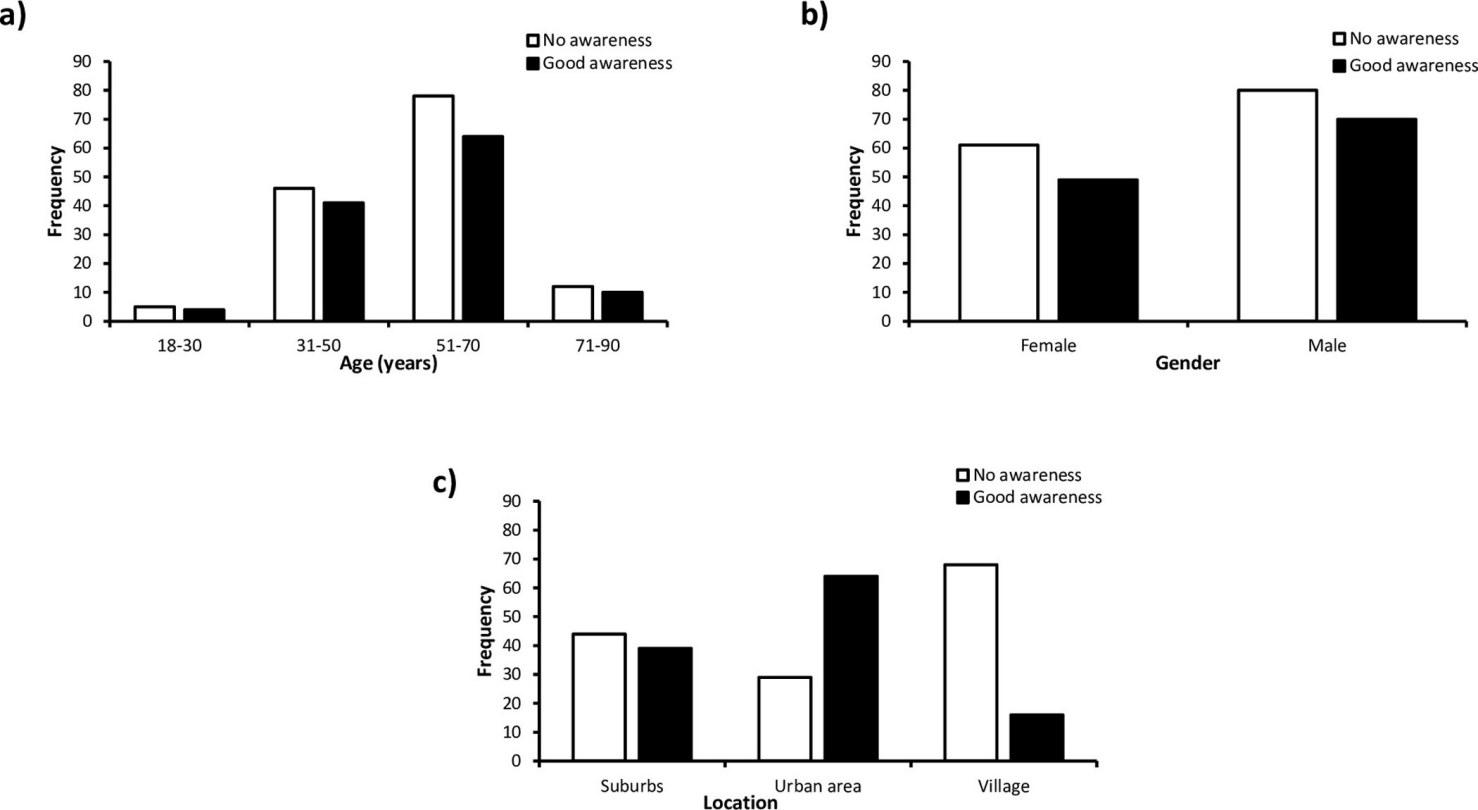
Frequency distribution of awareness of artificial sweeteners in foods in relation to key demographics. a) age group (p = 0.990); b) gender (p = 0.734); c) location setting (p<0.0001).

All the participant age groups studied reported a similar level of awareness of health issues linked to sweeteners except 51–70 years. In this age range, 78 people noted no awareness and 64 noted awareness of which foods contain artificial sweeteners (Fig 1A). Similarly, there were no significant associations noted between participant gender and awareness of sweeteners (Fig 1B). In contrast, there is a significant association noted between the location of participants and their awareness of sweeteners in food. The majority of participants from rural or village locations reported little awareness of sweeteners (80.95%) whereas more participants from urban or city areas noted good awareness of sweeteners in foods (68.82%) (Fig 1C).

### Changes in other habits

Finally, participants reported other changes in habits which they made pre- and post-diagnosis regarding smoking and chewing of tobacco (betel vine). Following diagnosis of diabetes, there was very little change noted in smoking with only 8 participants (3.08%) switching from a smoking to non-smoking habit. In contrast, there was a 16.92% reduction in the number of participants who reported chewing betel vine following diagnosis of diabetes. There was no significant association identified between the frequency of sugar or sweetener consumption and smoking or betel vine chewing habits (Table 5).

**Table 5:** Other lifestyle habits. The reported smoking or chewing of betel vine by participants pre- and post-diagnosis of diabetes. p = 0.951 and p = 0.184 respectively.

Category	Response	No. of participants	%
***Before diagnosis with diabetes***			
Smoking habit	Yes	61	23.46
	No	199	76.54
Betel vine (pan) habit	Yes	119	45.77
	No	141	54.23
***After diagnosis with diabetes***			
Smoking habit	Yes	53	20.38
	No	207	79.62
Betel vine (pan) habit	Yes	75	28.85
	No	185	71.15

## Discussion

Given the increasing number of the population with diabetes in Bangladesh, and the potential link with artificial sweetener consumption, our studies investigated whether there was a change in sweetener preference or awareness following diagnosis of diabetes and whether this is linked to key demographics or co-morbidities. Our findings indicate that daily sweetener consumption is significantly associated with hypertension as a co-morbidity, however within the population studied, there was significantly higher incidence of obesity and kidney disease in female diabetic patients versus male counterparts. Our study also demonstrates that awareness of sweeteners and their potential health impacts are significantly associated with location where those in urban and suburban areas expressed a better understanding than those living in rural areas. Taken together, these studies offer an insight into how diabetics in Bangladesh respond to artificial sweeteners and gives an indication of potential co-morbidities which may be associated with consumption of these non-nutritive food additives.

The demographics of patients with diabetes in developing countries is well understood with long-term prospective studies showing diabetes incidence increasing significantly from 40 years onwards and 55–69 years being most pre-disposed to having diabetes [29–32]. In our study, the prevalence of diabetes was concentrated on the older age group (51–70 years) which agrees with these literature and indicates that our sample population is representative of previously-described demographics. As the predominant form of diabetes, many previous studies focus on participants with type 2 diabetes. Indeed, the incidence of type 2 diabetes in our study was 85% however this statistic is lower than the 98% incidence of type 2 diabetes reported in Bangladesh in 2021 by the International Diabetes Federation [1]. The reason for the higher number of type 1 diabetes participants in our study versus those in the population is unclear but there are 5.38% of our study population who did not know which type of diabetes they suffered from. It is therefore possible that these participants had type 2 diabetes and would increase the statistic. This highlights that a focus on diabetes education and follow-up, post diagnosis, is very important in Bangladesh. From an international point of view, there is also an association between gender and prevalence of diabetes with slightly lower prevalence in women versus men [1]. This agrees with our findings that males were 57.69% of the participant population. There is, however, controversy in the literature regarding this. In Bangladesh, a large health survey showed that 52.1% of the diabetic population were female [33] which agrees with other studies indicating that non-European females were at greater risk from developing diabetes than males [34, 35] or, in Finland, finding that males are more prone to diabetes than females [36]. This gender prevalence seems to be highly dependent on global location as it is linked to genetic and epigenetic factors, nutrition, and a sedentary lifestyle, however these biological and psychosocial factors remain to be fully investigated in patients with diabetes in Bangladesh.

Our study focused on understanding whether patients switched from sugar to sweetener consumption following diagnosis. We observed that the vast majority of participants (95%) switched to artificial sweetener use after diagnosis of diabetes. Whilst this research has not previously been performed in Bangladesh, it is in keeping with global dietary recommendations provided to diabetic patients by medical professionals. Following diabetes diagnosis, other than switching from sugar to sweetener consumption, we also noted changes in lifestyle habits. Although not significant, there was a notable decrease in participants choosing to chew betel vine (a form of tobacco commonly used in Bangladesh) following diagnosis with diabetes however this was not mirrored with smoking. There is no literature to support the link between sweetener consumption and tobacco habits but betel vine chewing is strongly linked to increased obesity and cardiovascular disease in Eastern populations [37–39]. Therefore, it is possible that, following diagnosis of diabetes, patients actively choose to, or are recommended by their health provider to, give up betel vine as part of a positive health choice to improve their outcomes.

A key part of the study was to understand the awareness which diabetic patients in Bangladesh have of artificial sweeteners, both in terms of what food and drink they are found in and the potential health impact of consuming them. Most participants reported being unaware of any health effects of sweetener consumption and not checking food labels for sweetener presence before buying or consuming. In Bangladesh, food safety law has been a source of controversy for a long time as quality control observation and assurance is rare, irrespective of whether the supplier is a kitchen market or organised superstores [40]. Food adulteration and regulatory limitations of Bangladesh Standards and Testing Institution (BSTI) poses a potential threat to public health. Various chemicals such as carbide, formalin, ungraded textile colours and fertilisers have been identified in different food and vegetable products, raising the question of safety and the role of the BSTI [41]. Whilst the formation of the Bangladesh Food Safety Authority (BFSA) and Safe Food Law 2013 took control of this matter, food adulteration still remains a significant issue. A previous study based on a survey noted that, similar to our findings, people in Bangladesh do not actively report checking food labelling and will, instead, rely on food advertisement [40]. Therefore, there is a need to carefully consider the labelling of food products, specifically those linked to additives such as artificial sweeteners, as well as raising general awareness in the population to review these labels.

As part of studying awareness, we asked participants to self-report their awareness of artificial sweeteners in terms of health issues. Nearly 80% of the population we studied reported that they were not aware of any potential health concerns related to consuming sweeteners. This agrees with previous findings from India which reported over ¾ of the patients surveyed were unaware of any health hazards associated with artificial sweetener consumption [42]. Interestingly, this was significantly associated to location with participants from urban areas reporting a greater awareness than those living in rural areas. Given the fairly equal distribution of participants from urban, suburban and rural areas, the findings are indicative of a range of locations. Although this is surprising, given the global link between urbanization and increased incidence of diabetes [1], our findings agree with previous studies from the Bangladesh Demographic and Health Survey [43]. Of note, patients with diabetes in Bangladesh often report not seeking treatment or even monitoring blood glucose levels regularly with a large distinction between socioeconomic groups, and therefore location of dwelling [41, 44]. This is not a new concept and, across a range of nutrition types and disease states, it has been noted that reduced health awareness coupled with decreased access to healthcare systems is linked to greater incidence of disease and increased mortality [45–47]. More generally it reflects the limitations of the dissemination of scientific controversy among the population with a need to consider providing this level of education across the country.

An important dissemination aspect is the potential link between artificial sweeteners and diabetes or other health concerns. Our studies show that the majority of participants with diabetes reported 1, 2 or 3 comorbidities of which hypertension was the leading health concern highlighted. Hypertension is a common comorbidity among diabetic patients with the pathophysiology for the 2 conditions interlinked [32, 48, 49] therefore these findings can be expected however we also noted that obesity and kidney disease were significantly associated with female diabetics rather than male patients. Furthermore, our studies show that daily consumption of artificial sweeteners in the diet was significantly associated with hypertension in patients with diabetes. The molecular mechanism underlying artificial sweeteners and development of hypertension in patients with diabetes is unclear however it is worth noting that a recent study identified a direct association between consumption of the artificial sweeteners, aspartame, sucralose and aspartame, and increased risk of cardiovascular disease [50]. This suggests that there is a significant need to understand how artificial sweeteners link to hypertension, especially in vulnerable patients such as in diabetes.

Reducing the worldwide prevalence of diabetes, and the related healthcare and economic burden, requires long-term changes in public policy. Whilst these are largely centered around diagnosis, screening and treatment, consideration is needed on whether there is practical advice on artificial sweetener consumption which could make a difference in the healthcare of patients with diabetes, especially in developing countries. There is growing evidence in the literature indicating the potential negative impact of artificial sweetener consumption on healthcare which agrees with findings in the present study. It is, however, worth noting that there were limitations in this study; namely that studies were performed on a small number of the population and that findings were reliant on participants to self-report their dietary habits and that consideration of other related lifestyle factors (such as physical activity and general diet) was not included. Therefore, further study is recommended to more fully characterise this population of diabetic patients in Bangladesh and how artificial sweeteners can impact their health.

## Conclusions

Given the growing number of diabetic patients in Bangladesh, there is a significant need to carefully review treatment options as well as co-morbidities and lifestyle choices. In recent years, artificial sweetener consumption has been linked to the onset and exacerbation of diabetes. Our findings demonstrate a close association between sweetener consumption and hypertension in diabetic patients suggesting further health issues in these patients. We further show that the rurality of patient location mediates patient understanding of artificial sweeteners. We therefore propose that further research is needed to demonstrate how to increase education of sweetener consumption in the diabetic population in Bangladesh with the aim of reducing related co-morbidities and improving the quality of life for patients.

## Supporting information

S1 Table A list of the study questions posed to all participants. Once participants had answered screener questions, they were asked these questions in this order.
(DOCX)

S1 Checklist Inclusivity in global research checklist. (DOCX)

S2 Checklist (DOCX)
