# Supplementary material for: Investigating the use and awareness of artificial sweeteners among diabetic patients in Bangladesh
Source: PLoS One. 2023 Dec 13;18(12):e0295272. doi: 10.1371/journal.pone.0295272 (PMC10718416; doi:10.1371/journal.pone.0295272)
Supplement: S1 Table — Once participants had answered screener questions, they were asked these questions in this order. (DOCX) [file pone.0295272.s001.docx]

**Supplementary Table 1: A list of the study questions posed to all participants.** Once participants had answered screener questions, they were asked these questions in this order.

| **Question** | **Answer options** |
| --- | --- |
| What is your age (years)? | 18-30 |
|  | 31-50 |
|  | 51-70 |
|  | 71-90 |
|  | >90 |
| What is your identified gender? | Male |
|  | Female |
| Which of these most accurately describes where you live? | Village |
|  | Suburbs |
|  | Urban area |
| What type of diabetes do you have? | Type 1 diabetes |
|  | Type 2 diabetes |
|  | Gestational diabetes |
|  | I am not sure |
| How would you rate your knowledge of diabetes? | Good – I understand diabetes and what it means for my health |
|  | Fair – I have an idea of what diabetes is and how it affects me but could know more |
|  | Poor – I don’t understand diabetes at all |
| Do you currently have/or have you ever had any of the following health conditions? (select all relevant answers) | None of these |
|  | Hypertension |
|  | Obesity |
|  | Eye disease |
|  | Kidney disease |
|  | Chronic infection |
|  | Foot or leg problems |
|  | Dental problems |
|  | Sexual problems |
|  | Gastrointestinal problems |
|  | Other |
| Since being diagnosed with diabetes, how often do you eat sugary foods? | Never (I don't eat any) |
|  | Rarely (about 1-2 times per year) |
|  | Sometimes (about 1-2 times per month) |
|  | Often (about 1-2 times per week) |
|  | Daily (about 1-2 times per day) |
| Since being diagnosed with diabetes, how often do you replace sugar in your diet with artificial sweeteners? | Never (I don't use artificial sweeteners) |
|  | Rarely (about 1-2 times per year) |
|  | Sometimes (about 1-2 times per month) |
|  | Often (about 1-2 times per week) |
|  | Daily (about 1-2 times per day) |
| If you do use sweeteners, which ones do you typically use? (select any relevant answers) | I don’t use sweeteners |
|  | Sweet n Low |
|  | iLife Stevia Natural Sweeteners |
|  | MISTIN (Herbal Sweetening Powder) |
|  | Splenda No Calorie sweetener |
|  | Canderel Low Calorie sweetener |
|  | Silver Spoon sweetener |
|  | Equal sweetener |
|  | Huxol |
|  | Zerocal |
|  | Sugar free Gold |
|  | Xlear (XyloSweet) |
|  | Organic erythriol (Now Foods, Real Foods) |
|  | Organic monk fruit, xylitol |
|  | Other |
| Do you add artificial sweeteners to your tea/coffee? | Yes |
|  | No |
|  | I don’t drink tea or coffee |
| When you buy soft drinks, which type do you usually select? | With sugar |
|  | With low sugar |
|  | With no sugar |
| Are you aware of any foods which you eat which contain artificial sweeteners? | Yes – I am aware which foods contain sweeteners. |
|  | No – I am not aware which foods contain sweeteners |
| Do you usually check whether a product contains artificial sweeteners, e.g. by looking at the label? | Yes |
|  | No |
| Are you aware of health issues related to artificial sweetener usage? | Yes – I am aware of health issues. |
|  | No – I am not aware of health issues |
| Please answer the following questions based on **before**you were diagnosed with diabetes | |
| Did you smoke? | Yes |
|  | No |
| Did you chew battle-vine (Pan)? | Yes |
|  | No |
| Please answer the following questions based on **after**you were diagnosed with diabetes | |
| Do you smoke? | Yes |
|  | No |
| Do you chew betel-vine (Pan)? | Yes |
|  | No |
